# Effect of O-Antigen Chain Length Regulation on the Immunogenicity of *Shigella* and *Salmonella* Generalized Modules for Membrane Antigens (GMMA)

**DOI:** 10.3390/ijms22031309

**Published:** 2021-01-28

**Authors:** Gianmarco Gasperini, Maria Michelina Raso, Vanessa Arato, Maria Grazia Aruta, Paola Cescutti, Francesca Necchi, Francesca Micoli

**Affiliations:** 1 GSK Vaccines Institute for Global Health (GVGH) s.r.l, Via Fiorentina 1, 53100 Siena, Italy; gianmarco.x.gasperini@gsk.com (G.G.); maria-michelina.m.raso@gsk.com (M.M.R.); vanessa.x.arato@gsk.com (V.A.); maria-grazia.x.aruta@gsk.com (M.G.A.); francesca.x.necchi@gsk.com (F.N.); 2 Department of Life Sciences, University of Trieste, Via L. Giorgieri 1, Bdg C11, 34127 Trieste, Italy; pcescutti@units.it

**Keywords:** GMMA, O-antigen, vaccine, saccharide length, T-independent, *Shigella*, *Salmonella*

## Abstract

Recently, generalized modules for membrane antigens (GMMA) technology has been proposed as an alternative approach to traditional glycoconjugate vaccines for O-antigen delivery. Saccharide length is a well-known parameter that can impact the immune response induced by glycoconjugates both in terms of magnitude and quality. However, the criticality of O-antigen length on the immune response induced by GMMA-based vaccines has not been fully elucidated. Here, *Shigella* and *Salmonella* GMMA-producing strains were further mutated in order to display homogeneous polysaccharide populations of different sizes on a GMMA surface. Resulting GMMA were compared in mice immunization studies. Athymic nude mice were also used to investigate the involvement of T-cells in the immune response elicited. In contrast with what has been reported for traditional glycoconjugate vaccines and independent of the pathogen and the sugar structural characteristics, O-antigen length did not result in being a critical parameter for GMMA immunogenicity. This work supports the identification of critical quality attributes to optimize GMMA vaccine design and improve vaccine efficacy and gives insights on the nature of the immune response induced by GMMA.

## 1. Introduction

The O-antigen (OAg) moiety of lipopolysaccharide (LPS) has been recognized as a key target for protective immunity against several pathogens, including *Shigella* species and non-typhoidal *Salmonellae* (NTS) [1,2]. Recently, generalized modules for membrane antigens (GMMA) technology has been proposed as an alternative approach to traditional glycoconjugate vaccines for OAg delivery [3]. GMMA are outer membrane exosomes released from Gram-negative bacteria genetically engineered to increase blebbing (e.g., through deletion of the *tolR* gene) and reduce reactogenicity, usually by modifying the lipid A acylation pattern (e.g., through the deletion of the *htrB* and *msbB* genes) [4,5]. GMMA display the OAg in an outer membrane context and have been proven to be non-inferior compared to equivalent glycoconjugate vaccines in preclinical studies [6,7]. Moreover, GMMA can be produced at high yields and using a simple and robust process of manufacturing, potentially leading to affordable vaccines [8].

For traditional glycoconjugates, OAg length is well known to be a parameter that can impact the immune response induced [9]. For example, *Shigella sonnei* conjugates with low molecular-mass OAg fragments (average of 3.5 repeating units) induced significantly higher antibody levels in mice than the full-length OAg (approximately 29 repeating units) [10]. Additionally, a synthetic sequence of three repeating units of *Shigella flexneri* serotype 2a OAg conjugated to tetanus toxoid (TT) had higher immunogenicity and better protective efficacy in mice than conjugates with one or two repeating units [11]. Moreover, when tetra, octa, and dodecasaccharides from *Salmonella enterica* serovar Typhimurium OAg (corresponding to one, two, and three repeating units, respectively) were conjugated to bovine serum albumin (BSA), anti-LPS antibody titers increased with OAg length in mice and rabbits [12].

Polysaccharide length is also a parameter potentially impacting the type of immune response induced by glycoconjugate vaccines, which were traditionally developed to overcome T-independent immune responses [13,14]. Indeed, very long and highly repetitive surface structures like bacterial capsules and LPS are able to activate B lymphocytes through crosslinking of B-cell receptors (BCR) [15,16]. This results in the proliferation and differentiation of B lymphocytes and the production of antibodies. However, the absence of T-cell help results in a lack of affinity maturation, class-switching, and induction of immunological memory [17]. Importantly, when comparing typhoid conjugates made with short- or long-chain Vi polysaccharide, long–chain-conjugated Vi (165 kDa) induced a response in both wild-type and T-cell-deficient mice, while short-chain Vi (9.5 to 42.7 kDa) conjugates induced a response in wild-type mice but not in T-cell-deficient mice. This suggested that the response elicited by longer Vi chains was partially T-independent, consequently resulting in hyporesponsiveness [18]. Selection of optimal length in glycoconjugates can avoid detrimental effects on memory and subsequent boosting. However, the impact of OAg length on the magnitude and quality of the immune response induced by GMMA vaccines has not been elucidated in full.

*Shigella* and *Salmonella* OAg biosynthesis is dependent on the Wzx/Wzy pathway, and the degree of repeating unit polymerization (i.e., OAg size) is regulated by the Wzz family of proteins, responsible for unique polysaccharide modal lengths [19]. OAg repeating units can also be assembled into high molecular-mass capsules (Group 4 Capsules, G4C) if an additional G4C operon is present [20]. The introduction of mutations in the OAg locus or in the G4C operon results in a loss of specific polysaccharide populations on the bacterial surface [21,22], and consequently on GMMA [6].

We have previously performed studies in mice with *S. flexneri* serotype 6 GMMA differing in OAg length and found no major impact of the OAg length on the induced immune response [6]. Here, we extended the investigation to different OAg structures displayed by *S. sonnei*, *S. flexneri* 2a, and *S.* Typhimurium GMMA. The results from this study could be important in designing improved GMMA-based vaccines displaying OAg of optimal length.

## 2. Results

### 2.1. OAg Length Modulation through Genetic Engineering and Characterization of the Resulting GMMA

**Figure 1:**
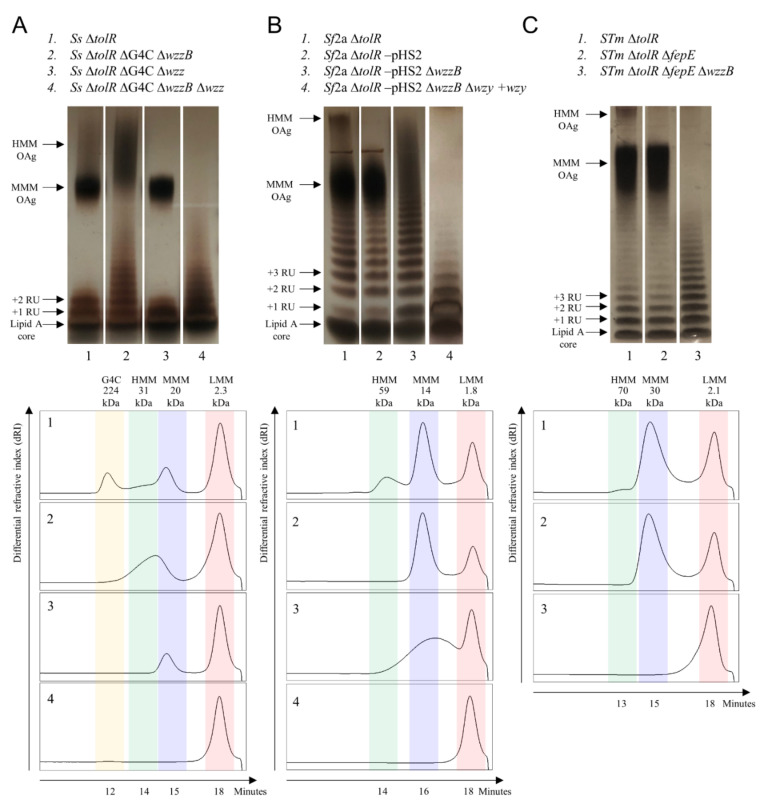
Silver staining of lipopolysaccharide (LPS) extracted from bacterial cells and HPLC–size exclusion chromatography (HPLC–SEC) profile differential refractive index (dRI) of sugar extracted from the corresponding generalized modules for membrane antigens (GMMA): (**A**) *S. sonnei*, (**B**) *S. flexneri* 2a, and (**C**) *S.* Typhimurium.

*S. sonnei* (*Ss*), *S. flexneri* 2a (*Sf*2a), and *S*. Typhimurium (*STm*) wild-type strains were engineered to obtain an hyperblebbing phenotype by removing the *tolR* gene. In order to evaluate the impact that OAg size could have on the immune response, the strains were further mutated by removing the genes reported in Table 1. The phenotype resulting from each mutation was analyzed by a silver-staining analysis of LPS extracted from bacterial cells and high-performance liquid chromatography–size exclusion chromatography (HPLC–SEC) analysis of OAg hydrolyzed with acetic acid from the corresponding GMMA (Figure 1).

*S. sonnei* Δ*tolR* LPS was characterized by one major sugar population of medium molecular mass (MMM), together with low molecular mass (LMM) OAg corresponding to few repeating units (Figure 1A). However, a high molecular mass (HMM) smear in the silver-stained gel suggested the occurrence of two closely unresolved polysaccharides (Figure 1A). This hypothesis was supported by the shoulder peak at 31 kDa in the HPLC–SEC chromatogram and the presence of two *wzz* homologues in the genome of *S. sonnei*. Moreover, an additional peak corresponding to a G4C polysaccharide was observed at 224 kDa in the HPLC–SEC chromatogram, as previously described [20]. To generate GMMA with more homogeneous polysaccharide populations, the *ept-etk* genes in the G4C operon were first removed in the *S. sonnei* Δ*tolR* strain to abolish the capsule formation (Figure 1A). Next, the chromosomal *wzzB* gene or the virulence plasmid *wzz* gene were removed in *S. sonnei* Δ*tolR* ΔG4C and yielded GMMA displaying HMM OAg or MMM OAg, respectively, together with LMM OAg (Figure 1A). Finally, removal of both *wzz* and *wzzB* in *S. sonnei* yielded GMMA displaying LMM OAg only, as previously observed for *S. flexneri* [6].

*S. flexneri* 2a LPS was characterized by two main sugar populations of different average molecular mass, together with LMM OAg (Figure 1B). The pHS2 plasmid (encoding FepE) or the chromosomal *wzzB* gene were removed in *S. flexneri* 2a Δ*tolR* to prevent HMM OAg or MMM OAg polymerization, respectively (Figure 1B). Contrary to what was observed in *S. flexneri* 6 and *S. sonnei*, removal of both pHS2-*fepE* and the *wzzB* gene in *S. flexneri* 2a Δ*tolR* yielded GMMA displaying long deregulated OAg chains (Figure 1B), meaning that even in the absence of the two OAg copolymerases, the Wzy polymerase is able to produce long OAg chains. Therefore, an alternative strategy was used to obtain GMMA displaying LMM OAg only. At first, the *wzy* gene was removed in *S. flexneri* 2a pHS2-cured Δ*tolR* Δ*wzzB* to obtain OAg chains composed by one single repeating unit, and subsequently complemented to partially restore the OAg polymerization (Figure 1B). The resulting strain yielded GMMA displaying LMM OAg only, with the majority of the OAg chains still composed by a single repeating unit, as verified by silver staining of extracted LPS (Figure 1B).

*S*. Typhimurium LPS was also characterized by two main sugar populations of different average molecular mass together with LMM OAg (Figure 1C), even though the HMM OAg population was minimal. The chromosomal *fepE* gene was removed in *S*. Typhimurium Δ*tolR* to prevent HMM OAg polymerization (Figure 1C). Next, the removal of the *wzzB* gene in *S*. Typhimurium Δ*tolR* Δ*fepE* yielded GMMA displaying LMM OAg only (Figure 1C).

The characterization of all the resulting GMMA is reported in Table 2. All GMMA had similar average particle size as measured by HPLC–SEC coupled with multiangle light scattering (MALS). The expected sugar composition was confirmed by high-performance anion exchange chromatography–pulsed amperometric detection (HPAEC–PAD) analysis in all mutants. The deletion of defined sugar populations impacted the sugar-to-protein ratio on the resulting GMMA. In particular, GMMA displaying LMM OAg only were characterized by the lowest OAg/protein (w/w) ratio. Consequently, the ability of each GMMA to induce IL-6 release in a monocyte activation test (MAT) with human peripheral blood mononuclear cells (PBMC) was higher for GMMA displaying LMM OAg only, when comparing the samples at the same OAg concentration. However, such difference is lost when comparing the samples at the same GMMA protein concentration, indicating that OAg length per se is not responsible for monocyte activation (Figure S1).

### 2.2. Immunogenicity Studies in Mice with GMMA Differing for OAg Length

Three in vivo studies were designed to compare the immunogenicity of GMMA displaying OAg with different lengths. All GMMA were compared in mice at the same OAg dose of 0.5 µg, and mice were immunized subcutaneously twice at 4-week intervals.

**Figure 2:**
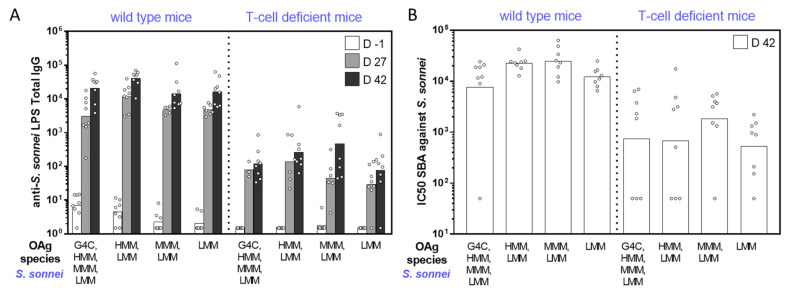
Immunogenicity in mice of *S. sonnei* GMMA differing for OAg length. Eight wild-type (CD1) and T-cell-deficient mice (nude CD1) per group were subcutaneously immunized on days 0 and 28, with 0.5 µg OAg dose. (**A**) Summary graphs of anti-LPS specific IgG reporting individual antibody levels (dots) and geometric mean with 95% confidence interval (bars). (**B**) Summary graph of serum bactericidal activity (SBA) titers against *S. sonnei* reporting individual IC50 levels (dots) and geometric mean (bars).

**Figure 3:**
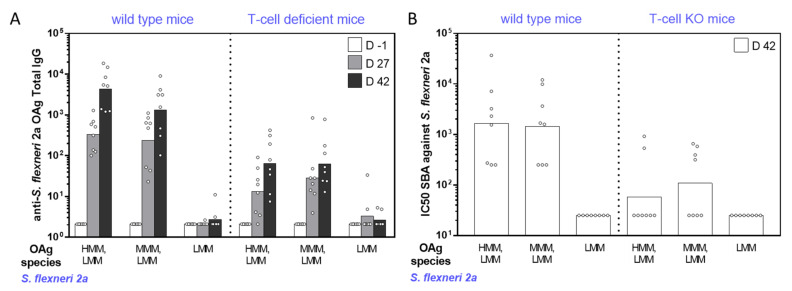
Immunogenicity in mice of *S. flexneri* 2a GMMA differing for sugar length. Eight wild-type (CD1) and T-cell-deficient mice (nude CD1) per group were subcutaneously immunized on days 0 and 28, with 0.5 µg OAg dose. (**A**) Summary graphs of anti-OAg specific IgG reporting individual antibody levels (dots) and geometric mean with 95% confidence interval (bars). (**B**) Summary graph of SBA titers against *S. flexneri* 2a reporting individual IC50 levels (dots) and geometric mean (bars).

**Figure 4:**
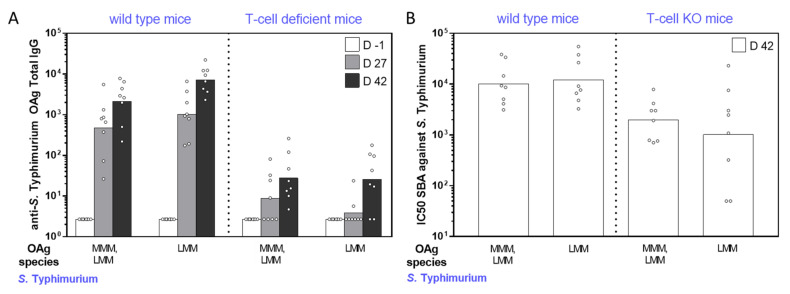
Immunogenicity in mice of *S.* Typhimurium GMMA differing for sugar length. Eight wild-type (CD1) and T-cell-deficient mice (nude CD1) per group were subcutaneously immunized on days 0 and 28, with 0.5 µg OAg dose. (**A**) Summary graphs of anti-OAg specific IgG reporting individual antibody levels (dots) and geometric mean with 95% confidence interval (bars). (**B**) Summary graph of SBA titers against S. Typhimurium reporting individual IC50 levels (dots) and geometric mean (bars).

Independent of the OAg length, *S. sonnei* GMMA induced high levels of anti-LPS specific IgG with no significant differences among the groups (Figure 2A). Similar results were obtained when comparing *S. flexneri* 2a and *S*. Typhimurium GMMA with all different constructs inducing high levels of anti-OAg specific IgG (Figure 3A and Figure 4A). However, *S. flexneri* 2a GMMA displaying LMM OAg only were not able to induce a significant anti-OAg IgG response (Figure 3A). Serum bactericidal activity (SBA) analysis of sera collected on day 42 confirmed the results obtained by enzyme-linked immunosorbent assay (ELISA) and proved the functional activity of sera raised against each GMMA (Figure 2B, Figure 3B, and Figure 4B).

The same GMMA were also compared in T-cell-deficient mice to evaluate their ability to induce a T-independent response according to the different OAg chain lengths. All GMMA, independent of OAg length, induced a significant response in T-cell-deficient mice (*p* < 0.03 comparing day -1 and day 42 ELISA units by nonparametric paired *t*-test), even though clearly lower than that induced in wild-type mice (Figure 2A, Figure 3A, and Figure 4A). Again, the SBA analysis of sera collected on day 42 from T-cell-deficient mice confirmed the results obtained by ELISA (Figure 2B, Figure 3B, and Figure 4B).

## 3. Discussion

In order to design effective vaccine candidates, it is important to verify the criticality of specific quality attributes. OAg length is one of the parameters that can affect the magnitude and quality of the immune response induced by OAg-conjugate vaccines [11,12,23,24]. GMMA can be characterized by very different OAg lengths as a result of pathogen-specific biosynthetic pathways leading to the expression of heterogeneous polysaccharide species with different degrees of polymerization. Therefore, further manipulation of the GMMA-producing strains to display homogeneous OAg populations with a defined optimal length could potentially lead to improved immunogenicity.

We have previously verified that OAg length does not play a major role on the immune response elicited by *S. flexneri* 6 GMMA and that even short OAg chains are able to induce a high and functional anti-OAg specific immune response, similar to that induced by long OAg chains [6]. In this study, we extended the investigation to *S. sonnei*, *S. flexneri* 2a, and *S*. Typhimurium GMMA, which all display OAg populations with different length and net sugar charge (zwitterionic sugars for *S. sonnei* and neutral sugars for *S. flexneri* 2a and *S.* Typhimurium, compared to negatively charged sugars for *S. flexneri* 6).

A panel of isogenic mutant strains was generated for *S. sonnei*, *S. flexneri* 2a, and *S*. Typhimurium to prevent the display of specific polysaccharide species on the bacterial surface and on the resulting GMMA. Mice immunogenicity studies confirmed that independent of OAg length, all GMMA induced comparable levels of functional anti-OAg responses when delivered at the same OAg dose, with the exception of *S. flexneri* 2a GMMA displaying LMM OAg. Indeed, they were not able to induce a significant anti-OAg IgG response in the conditions tested differently from GMMA displaying longer OAg chains. This was most likely due to the different strategy applied to obtain such a GMMA-producing strain which eventually displayed LMM OAg mainly composed of only one repeating unit. Previous studies have already suggested that a minimum of two repeating units of synthetic *S. flexneri* 2a OAg are required for optimal mimicry of OAg epitopes [25,26]. Polysaccharide length could also potentially impact the type of immune response induced by GMMA, particularly in terms of T-cell dependence. T-independent (TI) antigens are able to induce antibody responses in the absence of T-helper cells in vivo, as in the case of T-cell-deficient mice, and they can be divided into type 1 and type 2 antigens. Repetitive antigens such as bacterial polysaccharides are usually TI-2 antigens able to activate mature B lymphocytes via BCR crosslinking. On the contrary, polyclonal B-cell activators such as LPS are TI-1 antigens, able to cause proliferation and differentiation of mature and immature B lymphocytes via toll-like receptors (TLR) and independently of their BCR specificity [27].

Interestingly, despite OAg length, all GMMA induced significant and comparable IgG responses in T-cell-deficient mice. Although we cannot exclude that GMMA displaying shorter OAg chains might induce decreased direct B-cell activation compared to longer sugars, as previously observed in a dose-ranging study with *S. flexneri* 6 GMMA [6], our results indicate that, when presented on GMMA, even short OAg chains are able to elicit part of their response through direct activation of B-cells without involvement of T-cells. This could be explained by the particulate nature of GMMA as OAg delivery systems. Indeed, GMMA can be described as nanoparticles presenting highly organized and repetitive antigens (i.e., OAg) to the immune system. The multiplicity and geometry of antigen display can favor efficient BCR crosslinking, delivering strong activation signals to the B-cells independently from polysaccharide length [28]. Moreover, GMMA contain different TLR agonists, including lipid A and lipoproteins, able to provide synergistic TLR signaling to B-cells [5], as confirmed by MAT.

T-independent immune responses to bacterial polysaccharides are traditionally considered undesirable, as they are associated with a lack of affinity maturation, class-switching, and induction of immunological memory [14]. However, the differences observed between wild-type and T-cell-deficient mice clearly highlight a strong T-dependent component of the immune response induced by GMMA. Moreover, we have recently verified the ability of *S. sonnei* GMMA to boost the immune response in healthy European adults primed 2–3 years earlier [29]. Therefore, while polysaccharide length can dramatically impact the ability of glycoconjugate vaccines to induce T-independent responses [18], it might have a more limited role in the complex immunological response to GMMA [29].

From a practical point of view, the variability in OAg size expressed by bacteria that are used as the source for polysaccharide antigens represents a challenge for glycoconjugate vaccine development. Different bacterial strains display a wide range of OAg lengths that do not necessarily include the desired size for optimal immune stimulation. Therefore, additional sizing steps might be needed during purification to exclude suboptimal lengths from complex polysaccharide mixtures, thus increasing the overall process costs while decreasing the final yield [30]. In this study, we demonstrated that Gram-negative bacteria can be easily engineered to display defined OAg populations on GMMA particles. Importantly, GMMA not only can be used as vaccines per se but also serve as improved sources for purification of OAg of defined size for subsequent conjugation to carrier proteins [31,32].

In conclusion, OAg length does not seem to be a critical parameter for GMMA immunogenicity, in contrast to what has been reported for traditional glycoconjugate vaccines, and is independent from the pathogen and the sugar structural characteristics. However, attention must be paid to avoid the lack of the desired immune response as observed for *S. flexneri* 2a GMMA displaying LMM OAg only. This work investigates the criticality of a quality attribute (i.e., OAg length) to optimize GMMA design for use in vaccines and gives insights on the nature of the immune response induced by GMMA.

## 4. Materials and Methods

### 4.1. Bacterial Strains and Generation of Mutants

*S. sonnei* strain ATCC 25931, *S. flexneri* 2a strain 2457A, and *S*. Typhimurium strain 1418 (LT2 collection, University of Calgary) were chosen as parent strains and engineered to obtain the different mutants. The null mutations were obtained by replacing the genes of interest with an antibiotic resistance cassette by homologous recombination using a lambda red recombineering system [33]. In some cases, the antibiotic cassette was removed using the pCP20 plasmid, as previously described [33]. To maintain the OAg expression in *S. sonnei*, the virulence plasmid pSS carrying the OAg biosynthetic genes was stabilized by introduction of an antibiotic cassette (chloramphenicol resistance in the *msbB2* locus or *wzz* locus). For curing of the *fepE*-carrying plasmid pHS2 in *S. flexneri* 2a, the origin of replication of pHS2 was amplified and cloned into the pUC19 vector. The pUC19-pHS2ori vector was then used to cure pHS2 by plasmid incompatibility. For complementation of the *wzy* gene in *S. flenxeri* 2a, the *wzy* coding sequence and promoter were amplified and cloned into the pACYC-Duet vector. The list of all the bacterial strains generated and primers used is reported in Supplementary Table S1. The phenotype of each mutant was verified by a silver-staining analysis of LPS extracted from bacterial cells, as previously described [20].

### 4.2. GMMA Production and Characterization

*S. sonnei, S. flexneri* 2a, and *S*. Typhimurium GMMA were produced and purified as previously described [6]. The total protein content was estimated by bicinchoninic acid assay (BCA) using BSA as a reference. The total OAg amount and sugar composition were determined by HPAEC–PAD analysis, after performing acid hydrolysis directly on GMMA. In particular, the OAg amount was quantified based on the detection of repeating unit-specific monosaccharides (2-aminouronic acid for *S. sonnei*; rhamnose for *S. flexneri* 2a and *S*. Typhimurium), as previously described [34,35,36]. Purity and particle sizes were established by HPLC–SEC MALS, using TSK gel G6000PW + G4000PW columns (Tosoh Bioscience, Tokio, Japan) in series equilibrated in Phosphate Buffer Saline (PBS) [37]. The OAg extracted was characterized by HPLC–SEC (TSK gel 3000 PWXL column with TSK gel PWXL guard column equilibrated in 0.1 NaCl, 0.1 NaH_2_PO_4_, 5% CH_3_CN, Tosoh Bioscience, Tokio, Japan) with differential refractive index (dRI) detection using dextrans as the standards to estimate the molecular size distribution, as previously described [34,35]. The ability of each GMMA to stimulate IL-6 release from human PBMC was evaluated by MAT [38].

### 4.3. Mouse Studies

*S. sonnei*, *S. flexneri* 2a and *S*. Typhimurium GMMA differing in length were tested in mice. Female, five-week-old wild-type or T-cell-deficient CD1 mice were immunized subcutaneously (SC) with 200 µL of vaccine containing 0.5 µg OAg on days 0 and 28. For all studies, single sera were collected on day -1, 27, and 42. All animal studies were performed at Toscana Life Science Animal Care Facility under the animal project 479/2017-PR 09/06/2017 approved by the Italian Ministry of Health. The studies were ethically reviewed and carried out in accordance with European Directive 2010/63/EEC, the GSK policy on the Care, Welfare and Treatment of Animals, and local animal welfare guidelines in Toscana Life Sciences facilities under Italian authorization. All sera were analyzed by ELISA [39,40] for anti-*S. sonnei* LPS total IgG using *S. sonnei* LPS as plate-coating antigen (at 0.5 µg/mL concentration in PBS), anti-*S. flexneri* 2a OAg total IgG using *S. flexneri* 2a OAg as coating antigen (at 0.5 µg/mL concentration in Carbonate buffer), or anti-*S*. Typhimurium OAg total IgG using *S*. Typhimurium OAg as coating antigen (at 5 µg/mL concentration in Carbonate buffer). All coating antigens were purified in house as previously described [39,41]. Single sera collected on day 42 were also assayed in SBA based on luminescent readout against *S. sonnei, S. flexneri* 2a, or *S*. *Typhimurium* wild-type strains, as previously described [6,42].

### 4.4. Statistical Analysis

Analysis was performed using GraphPad Prism 7 (La Jolla, California, United States). The Mann–Whitney U-test was used to compare two groups and a Kruskal–Wallis analysis with post-hoc Dunn’s test to compare multiple groups. The Wilcoxon matched-pairs signed rank two-tailed test was used to compare results from the same group at different time points (day -1 vs. day 42).

## Figures and Tables

**Table 1:** Mutations introduced for hypervesiculation and O-antigen (OAg) length regulation.

Gene Deleted	Gene Function	Resulting Phenotype
*ept-etk*	Part of the G4C operon needed for capsule assembly and export	Lack of G4C formation
*wzz* (pSS)	pSS plasmid-borne OAg co-polymerase, responsible for HMM OAg polymerization in *S. sonnei*	Lack of HMM OAg formation
*fepE* (pHS2)	pHS2 plasmid-borne OAg co-polymerase, responsible for HMM OAg polymerization in *S. flexneri*	Lack of HMM OAg formation
*fepE*	Chromosomal encoded OAg co-polymerase, responsible for HMM OAg polymerization in *S*. Typhimurium	Lack of HMM OAg formation
*wzzB*	Chromosomal encoded OAg co-polymerase, responsible for MMM OAg polymerization in *S. sonnei, S. flexneri* and *S.* Typhimurium	Lack of MMM OAg formation in *S. sonnei* and *S.* Typhimurium; De-regulation of OAg polymerization in *S. flenxeri 2a*
*wzy*	OAg polymerase, responsible for the polymerization of the repeating units in *S. sonnei* and *S. flexneri*	Formation of OAg composed by 1 single repeating unit

**Table 2:** Analytical characterization of GMMA displaying OAg populations of different length.

GMMA	Mutations	OAg Length	Total Sugar/Protein *w*/*w* Ratio	Size 2 × Rw nm
*S. sonnei*	*ΔtolR*	G4C, HMM, MMM, LMM OAg	0.34	87.0
	*ΔtolR* Δ*G4C* Δ*wzzB*	HMM, LMM OAg	0.35	90.3
	*ΔtolR* Δ*G4C* Δ*wzz*	MMM, LMM OAg	0.18	91.4
	*ΔtolR ΔG4C Δwzz ΔwzzB*	LMM OAg	0.14	90.8
*S. flexneri 2a*	*ΔtolR*	HMM, MMM, LMM OAg	0.58	89.6
	*pHS2-cured ΔtolR*	MMM, LMM OAg	0.53	91.4
	*pHS2-cured ΔtolR ΔwzzB*	De-regulated OAg	0.40	93.6
	*pHS2-cured ΔtolR ΔwzzB Δwzy pACYC-wzy*	LMM OAg	0.17	81.0
*S.* Typhimurium	*ΔtolR*	HMM, MMM, LMM OAg	1.35	64.4
	*ΔtolR ΔfepE*	MMM, LMM OAg	1.42	66.2
	*ΔtolR ΔfepE ΔwzzB*	LMM OAg	0.44	62.2

## Data Availability

Data is contained within the article or supplementary material.

## Supplementary Materials

The following are available online at https://www.mdpi.com/1422-0067/22/3/1309/s1, **Figure S1**.: Monocyte Activation Test (MAT), **Table S1**.: List of bacterial strains and primers used in this study.
