# Supplementary material for: Effect of O-Antigen Chain Length Regulation on the Immunogenicity of Shigella and Salmonella Generalized Modules for Membrane Antigens (GMMA)
Source: Int J Mol Sci. 2021 Jan 28;22(3):1309. doi: 10.3390/ijms22031309 (PMC7865430; doi:10.3390/ijms22031309)
Supplement: Supplementary file 1 [file ijms-22-01309-s001.pdf]

International Journal of
Molecular Sciences

MDPI

Article

Effects of Chain Length Regulation of O-Antigen Polysaccharides on the Immunogenicity of Generalized Modules for Membrane Antigens (GMMA)-Based Vaccines

Gianmarco Gasperini [1], Maria Michelina Raso [1,2], Vanessa Arato [1], Maria Grazia Aruta [1], Paola Cescutti [2], Francesca Necchi [1] and Francesca Micoli [1,*]

[1] GSK Vaccines Institute for Global Health (GVGH) s.r.l, Via Fiorentina 1, 53100 Siena, Italy; gianmarco.x.gasperini@gsk.com (G.G.); maria-michelina.m.raso@gsk.com (M.M.R.); vanessa.x.arato@gsk.com (V.A.); maria-grazia.x.aruta@gsk.com (M.G.A.); francesca.x.necchi@gsk.com (F.N.)

[2] Department of Life Sciences, University of Trieste, Via L. Giorgieri 1, Bdg C11, 34127 Trieste, Italy; pcescutti@units.it

* Correspondence: francesca.x.micoli@gsk.com; Tel.: +39-0577-539087

Supplementary Materials

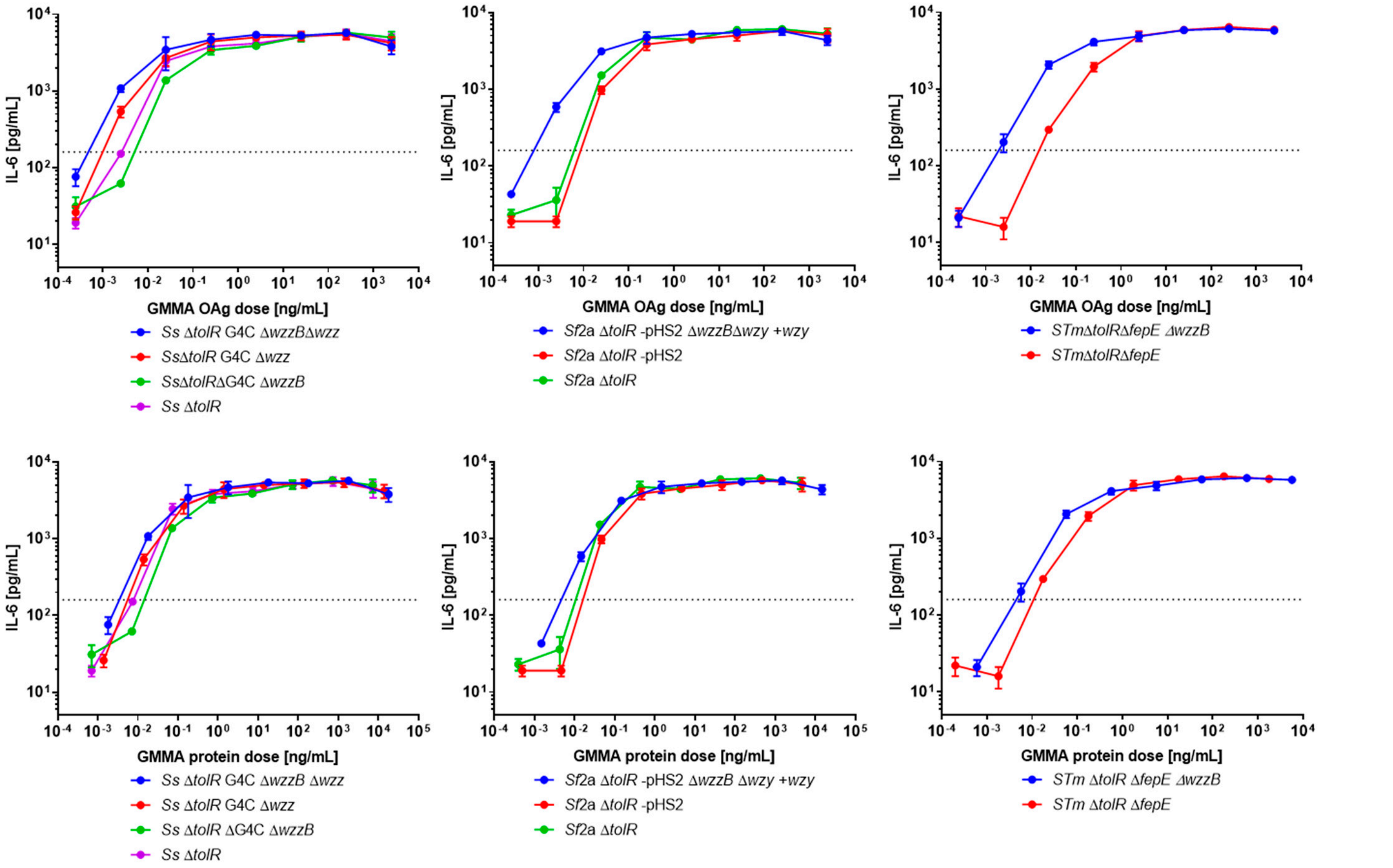

Figure S1. Monocyte Activation Test (MAT): IL-6 released by human PBMC (Biopedric) after stimulation with each GMMA, normalized based on the OAg dose (upper panels) or the GMMA protein dose (lower panels). The dashed horizontal black line represents a 10-fold increase over background.

Table S1. List of bacterial strains and primers used in this study.

**Bacterial Strains**	
**Name**	**Genotype**
S. sonnei wild type	*S. sonnei 53G ΔvirG::cm*
S. sonnei ΔtolR	*S. sonnei* ATCC 25931 *ΔtolR::kan ΔmsbB2::cm*
S. sonnei ΔtolR ΔG4C ΔwzzB	*S. sonnei* ATCC 25931 *ΔtolR::erm ΔmsbB2::cm Δept-etk::tet ΔwzzB::kan*
S. sonnei ΔtolR Δ*G4C* Δ*wzz*	*S. sonnei* ATCC 25931 *ΔtolR::kan Δept-etk::tet Δwzz::cm*
S. sonnei ΔtolR ΔG4C Δwzz ΔwzzB	*S. sonnei* ATCC 25931 *ΔtolR::erm Δept-etk::tet Δwzz::cm ΔwzzB::kan*
S. flexneri 2a wild type	*S. flexneri* 2a 2457A
S. flexneri 2a *ΔtolR*	*S. flexneri* 2a 2457A *ΔtolR::frt*
S. flexneri 2a *pHS2-cured ΔtolR*	*S. flexneri* 2a 2457A *ΔtolR::frt pUC19-pHS2ori*
S. flexneri 2a *pHS2-cured ΔtolR ΔwzzB*	*S. flexneri* 2a 2457A *ΔtolR::frt ΔwzzB::frt pUC19-pHS2ori*
S. flexneri 2a *pHS2-cured ΔtolR ΔwzzB Δwzy*	*S. flexneri* 2a 2457A *ΔtolR::frt ΔwzzB::frt Δwzy::kan pUC19-pHS2ori*
S. flexneri 2a *pHS2-cured ΔtolR ΔwzzB Δwzy pACYC-wzy*	*S. flexneri* 2a 2457A *ΔtolR::frt ΔwzzB::frt Δwzy::kan pUC19-pHS2ori pACYC-Duet_wzy*
S. Typhimurium wild type	*S. enterica serovars Typhimurium isolate D23580*
S. Typhimurium Δ*tolR*	*S. enterica serovars Typhimurium isolate 1418 ΔtolR::frt*
S. Typhimurium Δ*tolR* Δ*fepE*	*S. enterica serovars Typhimurium isolate 1418* Δ*tolR* Δ*fepE::cm*
S. Typhimurium Δ*tolR* Δ*fepE* Δ*wzzB*	*S. enterica serovars Typhimurium isolate 1418* Δ*tolR* Δ*fepE::cm* Δ*wzzB::kan*

**Primers**	
**Name**	**5′-3′ Sequence**
Shigella tolR KO Fwd (*aph/frt*)	ACCGCCAGGCGTTTACCGTTAGCGAGAGCAACAAGGGGTAAGCCATGGCC**GTGTAGGCTGGAGCTGCTTC**
Shigella tolR KO Rev (*aph/frt*)	ACCCGCTCTCTTTCAAGCAAGGGAAACGCAGATGTTTAGATAGGCTGCGT**CATATGAATATCCTCCTTAG**
Shigella tolR KO Fwd (*erm*)	ACCGCCAGGCGTTTACCGTTAGCGAGAGCAACAAGGGGTAAGCCATGGCC**AGAGTGTGTTGATAGTGCAGTATC**
Shigella tolR KO Rev (*erm*)	ACCCGCTCTCTTTCAAGCAAGGGAAACGCAGATGTTTAGATAGGCTGCGT**ACCTCTTTAGCTTCTTGGAAGCT**
Salmonella tolR KO Fwd (*aph/frt*)	CCAGGCGTTTACCGTAAGCGAAAGCAACAAGGGGTAA**GTGTAGGCTGGAGCTGCTTC**
Salmonella tolR KO Rev (*aph/frt*)	CCTGTTACTCGCCGTCTTTCAAGCCAACGGGACGCAGACT**CATATGAATATCCTCCTTAG**
msbB2 KO Fwd (*cat*)	TAAAATATTAATGATGATTATGGTAGGGGCATTCGCACTAAATAATGAAA**GTGTAGGCTGGAGCTGCTTC**
msbB2 KO Rev (*cat*)	ACAACTAGTGGAAATACCTGTACTTTATAATTTCAAGGGTACGGGTCCGC**CATATGAATATCCTCCTTAG**
Shigella wzzB KO Fwd (*aph/frt*)	AGAGTAGAAAATAATAATGTTTCTGGGCAAAACCATGACCCGGAACAGAT**GTGTAGGCTGGAGCTGCTTC**
Shigella wzzB KO Rev (*aph/frt*)	CTTCGCGTTATAATTACGCAGAGCGTTACGCCCCAGCACAATCCCCGCGC**CATATGAATATCCTCCTTAG**
Salmonella wzzB KO Fwd (*aph*)	GATTGATTTGATTGAGTTATTGCTACAGTTATGGCGTGGGAAGATGACCA**GTGTAGGCTGGAGCTGCTTC**
Salmonella wzzB KO Rev (*aph*)	TCCCGGCACCGATCATCCCACCCAGCAATACAGCCAGCACAAGGGTAATG**CATATGAATATCCTCCTTAG**
Salmonella fepE KO Fwd (*cat*)	TTCACTGCCGCCCGCCAACAGTCATGAAATCGATTTGTTTAGCCTTATAG**GTGTAGGCTGGAGCTGCTTC**

Salmonella fepE KO Rev (*cat*)	CCATCGCGTGACGCAGTAATACGCCGCCGCAGGCCATCATACCGCCAATC**CATATGAATATCCTCCTTAG**
ept-etk KO Fwd (*tet*)	TTACTCTTTCTCGGAGTAACTATAACCGTAATAGTTATAGCCGTAACTGT**CACCTGAAGTCAGCCCC**
ept-etk KO Rev (*tet*)	AATATCTATCCCGTCACGCCAGGATTGATTGATCAGTTGCGCGCCAAACC**CTCCAATTCTTGGAGTGGT**
wzz KO Fwd (*cat*)	TCAAAAGCATCTGAACCACAACAGACCCCTTATCTGATCCCGCAAGGGGT**GTCTTGAGCGATTGTGTAGG**
wzz KO Rev (*cat*)	ATTAACCAAAAGAGTATTAACATTTTGACCAAACTCATCAAGACTTAATG**TCCTCCTTAGTTCCTATTCC**
wzy KO Fwd (*aph*)	TTTTGCTCCAGAAGTGAGGTTATTACTAATTTGGATATTTTCTATAGAAA**GTGTAGGCTGGAGCTGCTTC**
wzy KO Rev (*aph*)	TATTGGTGGTGGTGGAAGATTACTGGAGCCATTGGGAATATTCCCTTTGC**CATATGAATATCCTCCTTAG**
pHS2-Ori Fwd XbaI	TTGAGATCATTCCCTTACAGCCG
pHS2-Ori Rev XmaI	CTGAACGCGATTCTGCCG
wzy Fwd BamHI	CGC**GGATCC**ACGGCTTGGTTTGGTGAGAA
wzy Rev XhoI	CCG**CTCGAG**CTTCGGCCTTGACCAAAAGC
